# The Clinical Utility of Albumin with Sequential Organ Failure Assessment (SOFA) in Improving 30-Day Mortality Prediction in Patients with Infection in the Emergency Department

**DOI:** 10.3390/jcm12247676

**Published:** 2023-12-14

**Authors:** Gianni Turcato, Arian Zaboli, Serena Sibilio, Michael Mian, Francesco Brigo

**Affiliations:** 1 Department of Internal Medicine, Intermediate Care Unit, Hospital Alto Vicentino (AULSS-7), 36014 Santorso, Italy; gianni.turcato@yahoo.it; 2 Innovation, Research and Teaching Service (SABES-ASDAA), Teaching Hospital of the Paracelsus Medical Private University (PMU), Via A. Volta 5, 39049 Bolzano, Italy; zaboliarian@gmail.com (A.Z.); michael.mian@sabes.it (M.M.); 3 Department of Emergency Medicine, Hospital of Merano-Meran (SABES-ASDAA), 39012 Merano-Meran, Italy; serena.sibilio@sabes.it; 4 Lehrkrankenhaus der Paracelsus Medizinischen Privatuniversität, 5020 Salzburg, Austria; 5 College of Health Care-Professions Claudiana, 39100 Bozen, Italy

**Keywords:** infection, SOFA, sepsis, emergency medicine, albumin, Sequential Organ Failure Assessment, implementation, emergency department

## Abstract

Background: The Sequential Organ Failure Assessment (SOFA) score is currently the primary prognostic tool used in patients with infections to predict sepsis and mortality, although its predictive role remains debated. Serum albumin values have been recently found to correlate with the severity of sepsis. The purpose of this study is to evaluate the clinical usefulness of albumin dosage on SOFA score prediction in infected patients. Methods: This prospective single-centre observational study was performed in 2021. We used the net reclassification improvement (NRI) technique to evaluate the additional prognostic value of serum albumin used together with the SOFA score in infected patients. The discriminatory abilities of the SOFA score alone, of albumin levels alone, and of the albumin levels together with (but not incorporated into) the SOFA score was evaluated by comparing the area under the curve of the corresponding receiver operating characteristic (ROC) curves. Results: We included 949 patients with an infectious status; 8.9% (84/949) died within 30 days of ED admission. The AUROC for the SOFA score was 0.802 (95% CI: 0.756–0.849) and the albumin level was 0.813 (95% CI: 0.775–0.852). The NRI found that serum albumin improved SOFA score predictions of 30-day mortality by 24.3% (*p* < 0.001), yielding an AUROC of 0.881 (95% CI: 0.848–0.912; *p* < 0.001). Conclusions: Using serum albumin values together with the SOFA score can improve prognostic prediction in patients with infections evaluated in the ED.

## 1. Introduction

Infection-related symptoms are common reasons for admission to the emergency department (ED) [1,2,3,4]. Infectious states are a diverse group of clinical disorders that range from uncomplicated, non-severe infections that may not require evaluation in the ED to serious, often life-threatening conditions with a high risk of poor outcomes [1,2,3,4,5]. Sepsis and septic shock, in particular, are responsible for over 30% of all in-hospital mortality and cause 11 million deaths annually [3,6]. Only a few patients with infections who require hospitalization have severe haemodynamic impairment when they arrive in the ED [1,7]. Others appear clinically stable despite persistent microvascular and tissue changes that, if not discovered early, could lead to death [1,7,8].

The prognostic assessment and identification of the actual evolutionary risk of patients with infections remain a clinical challenge, since the available instruments do not seem to reach safe predictive levels [7,9]. Among these, the most commonly used is the Sequential Organ Failure Assessment (SOFA) score [10]. Originally developed to assess organ failure severity in critically ill patients in intensive care units (ICUs), it is now also used to assess the organ dysfunction in ED patients with an infection for supporting the diagnosis of sepsis and septic shock [11,12]. However, the SOFA score may be less accurate in the ED than in the ICU [12,13], possibly because patients with infections in the ED frequently present with potentially serious early pathophysiological processes but have not yet developed significant organ failure, and the SOFA score elements better reflect real organic damage than early organic tissue failure [12].

Serum albumin levels have been recently found to predict the severity of sepsis [8,14]. The decrease in albumin, which is associated with increased mortality, appears to correlate with the presence and severity of inflammation-induced microvascular changes that contribute to tissue hypoxia prior to the development of organ damage [8,14,15]. Patients with infections who present in the ED with a low albumin level, particularly those who are hemodynamically stable, may be at a higher evolutionary risk of death [8,14,15]. As a result, the prognostic role of this early indicator used together with the SOFA score may improve its suitability for predicting late-stage organ damage, complementing its determination of early tissue damage to improve overall prognostic prediction in patients with infections.

In the study, we aim to investigate whether the prognostic value of albumin used together with the SOFA score enhances its ability to predict 30-day mortality in patients with infectious status when they arrive in the ED.

## 2. Methods

### 2.1. Study Design and Setting

This observational, single-center study was performed between 1 January and 31 December 2021 at the ED of the Merano Hospital (Italy; 53,000 accesses in 2021). This was a secondary analysis of data collected in a previous study performed at the same ED [14].

### 2.2. Patients

This real-world study was performed in the ED setting. It included all patients aged ≥ 18 years who were admitted to the ED with a suspected infected status.

Currently, there is no clear definition for an ‘infected patient’ at the access in the ED [14]. For this reason, the choice of considering patients affected by an infection and to include them in the study was made by the ED physician. Prior to the start of the study, the physicians received a specific training, during which the study was explained, and it was clarified how to consider and evaluate patients with suspected infection. It was recommended to carefully evaluate the clinical presentation, the results of laboratory and radiological investigations, and to consider a patient as infected only if an infection was confirmed by specific investigations. However, due to the lack of an unequivocal definition of infection and of a clear methodology to identify it, the decision to enroll the patient in the study was left to the evaluating physician. The patient’s attending physician confirmed or excluded their infection diagnosis at the completion of the clinical instrumental examination in the ED. Patients who were not determined to be infected following the clinical instrumental assessment were excluded. Other exclusion criteria included (a) severe hepatic dysfunction with a total serum bilirubin level > 30.0 mg/dL, (b) AIDS or pregnancy, (c) administration of human albumin in the three weeks preceding the onset of infection, (d) admission to the ED within 15 days of a previous hospitalization or operation, (e) COVID-19 infection, (f) patients with coma or unstable clinical status. Of note, considering the peculiarities of our healthcare setting, patients do not receive any further treatment before accessing the hospital, unless they have an urgent and unstable clinical status, which would nonetheless prevent them from providing the consent to participate to the study.

All patients enrolled in this study consented to participation; non-consent led to their exclusion. 

### 2.3. Study Protocol

At the first ED assessment of patients with an infection, a battery of blood tests was performed. It included complete blood count with leucocyte differential, serum electrolyte levels, renal function, liver function, serum albumin level, C-reactive protein (CRP) level, total bilirubin level, coagulation status, and arterial blood gas. 

During the assessment, various demographic and clinical data were obtained, including sex, age, medical history, systolic blood pressure, diastolic blood pressure, respiratory rate, heart rate, capillary oxygen saturation, and cognitive impairment. In addition, the patients’ Charlson Comorbidity Index (CCI), National Early Warning Score (NEWS), and SOFA score were recorded [16,17].

A 3 mL blood sample was collected from a peripheral vein to determine the serum albumin level. The collected blood sample was then centrifuged, and the serum was separated. Serum albumin levels were measured using bromocresol green colorimetry with an Alinity ci-series analyzer (Abbott). The serum albumin levels are reported as grams per deciliter (g/dL).

All these investigations were performed during the patient’s initial evaluation in the ED. 

### 2.4. Outcome

This study’s primary outcome was the patient’s death within 30 days of their initial evaluation in the ED. Mortality was determined using death records or by contacting the registry office directly.

### 2.5. Statistical Analysis

Continuous variables are presented as the mean and standard deviation (SD) or the median and interquartile range (IQR), depending on the underlying distribution. Univariate comparisons were performed with Student’s *t*-test, the Mann–Whitney test, or the Kruskal–Wallis test as appropriate. Categorical variables are reported as the percentage and total number of events and were compared using Fisher’s exact test or the Chi-square test.

The correlation between comorbidity scores (CCI), acuity (NEWS) albumin and SOFA were investigated using Pearson or Spearman correlation according to their appropriateness. The choice of Pearson or Spearman correlation was made after evaluating the underlying variable distribution using the Shapiro–Wilk test.

We calculated the area under the receiver operating characteristic (AUROC) curve for 30-day mortality to assess the discriminatory ability of the SOFA score alone, of serum albumin levels alone, and of the albumin levels together with (but not incorporated into) the SOFA score. We compared their discriminatory abilities by comparing the AUROC curves.

AUROC values are reported with 95% confidence intervals (CIs).

Using net reclassification improvement (NRI), the potential improvement in risk stratification of the albumin levels together within the SOFA score was investigated [18,19]. Patients were divided into four a priori risk groups based on their absolute risk percentage: 5%, 5–15%, 15–30%, and >30%. The NRI explored whether the prognostic utility of the albumin levels used together with (but not incorporated into) the SOFA score can improve the classification of patient and control outcomes, moving them up or down in the risk categories. The NRI represents the unweighted sum of these improvements [18,19].

Decision curve analysis (DCA) is a simple method of assessing the clinical benefit of predictive models and formulating better clinical strategies. The DCA provides information on a range of threshold probabilities for patient risk outcomes obtained by using the albumin level together with the SOFA score as compared to normal clinical management.

All tests were two-tailed and *p*-values < 0.05 were considered statistically significant. Statistical analysis was conducted using STATA 16.0 statistical software (StataCorp, College Station, TX, USA).

### 2.6. Ethical Statement

This study was conducted according to the Declaration of Helsinki and approved by the local ethics committee (Comitato etico per la sperimentazione clinica dell’Alto Adige; approval number: 94-2020).

## 3. Results

This study included 949 patients with infectious status [12]. Their median SOFA score was 1 (0–3); 51.9% of the patients (493/949) had a SOFA score of 0–1, 28.3% (269/949) had a SOFA of 2–3, and 19.7% (187/949) had a SOFA score ≥ 4 (Table 1).

Table 1 shows the characteristics of the patients. Patients in the most severe SOFA group were older and more likely to suffer from chronic diseases. The CCI increased progressively as the SOFA score category increased (3.1 vs. 4.6 vs. 5.7; *p* < 0.001), showing a statistically significant positive correlation (Pearson’s correlation coefficient = 0.377, *p* < 0.001). The evaluation of vital signs also showed that the more severe SOFA categories had more altered vital signs. The median NEWS score increased from 2 (1–3) in patients with SOFA scores of <2 to 4 (2–6) in patients with SOFA scores of 2–3, and to 7 (4–10) in patients with SOFA scores of >3 (*p* < 0.001). NEWS scores and SOFA scores were positively correlated (Spearman’s correlation coefficient = 0.542, *p* < 0.001). The mean serum albumin level was 3.92 (0.47) g/dL in patients with SOFA scores of <2; 3.62 (0.51) g/dL in patients with SOFA scores of 2–3; and 3.26 (0.51) g/dL in patients with SOFA scores of ≥4 (*p* < 0.001). Finally, serum albumin levels were inversely correlated with SOFA scores (Pearson’s correlation coefficient = −0.494, *p* < 0.001).

Overall, 8.9% of the patients (84/949) died within 30 days of their ED evaluation. Table 2 shows the variables associated with 30-day mortality. The patients who died within 30 days were older and had more comorbidities. The mean CCI was 3.8 (2.9) for surviving patients and 6.4 (2.1) for patients who died within 30 days (*p* < 0.001). The median SOFA score was 1 (0–3) for surviving patients and 4 (3–6) for patients who died within 30 days (*p* < 0.001). The mean serum albumin level on arrival in the ED was 3.76 (0.52) g/dL for surviving patients and 3.09 (0.46) g/dL for patients who died within 30 days (*p* < 0.001).

**Figure 1:**
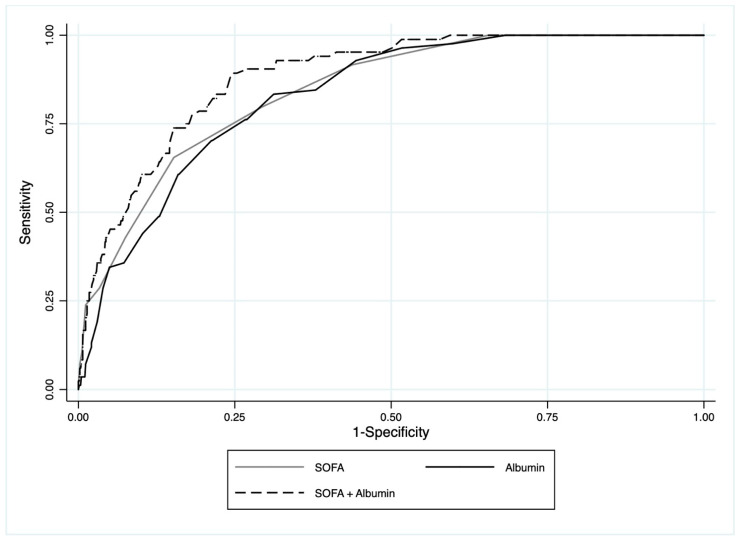
The ROC curves for 30-day mortality. The black line represents serum albumin, the grey line represents the SOFA score, and the black dashed line represents albumin values used together with the SOFA score.

Regarding discriminatory ability, the SOFA score had an AUROC of 0.843 (95% CI: 0.805–0.880), while the serum albumin level had an AUROC of 0.829 (95% CI: 0.792–0.867). The prognostic utility of the serum albumin level together with the SOFA score resulted in a higher predictive ability, with an AUROC of 0.882 (95% CI: 0.851–0.913; Figure 1). A significant difference was found between the three ROCs (*p* < 0.001). The results of the NRI are reported in Table 3. By evaluating the serum albumin levels together with the SOFA score, 11.9% of patients (10/84) who died within 30 days following their ED examination were correctly reclassified. It also resulted in a higher risk reclassification for 26.2% (22/84) of these patients and a lower risk reclassification for 14.3% (12/84) of them. Furthermore, it allowed for the accurate categorization of 11.8% (102/865) of survivors. It resulted in the accurate lower risk reclassification for 19.4% (168/865) vs. an incorrect higher risk reclassification for 7.1% (61/865).

**Figure 2:**
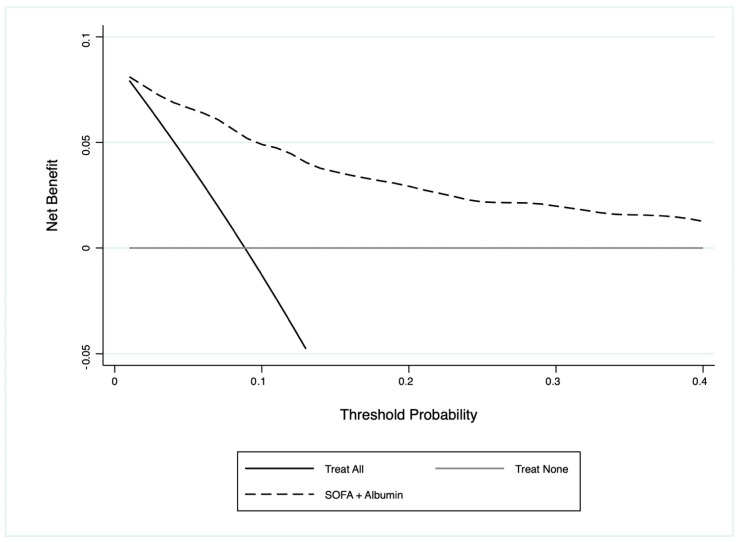
DCA for determining the net clinical benefit from using albumin values together with the SOFA score (black dashed line) in patients with infections in the ED. The *x*-axis indicates the threshold probability for adverse cardiac events, and the *y*-axis indicates the net benefit. The black line assumes that all the patients would have that outcome, whereas the grey line assumes that none would have that outcome. The dashed black line represents the net clinical benefit of using albumin values together with the SOFA score.

Overall, the serum albumin levels used together with the SOFA score improved its prediction of 30-day mortality by 24.3% (*p* < 0.001). The potential net clinical benefit of the serum albumin levels used together with the SOFA was assessed using DCAs (Figure 2). Its inclusion provided a net clinical benefit greater than the standard assessment for a wide range of threshold probabilities. With a threshold probability of 5–15%, the albumin levels used together with the SOFA score resulted in a net clinical benefit of 2–6%, implying the possibility of detecting up to 6 more patients at risk of 30-day mortality within 100 infected individuals assessed in the ED (Figure 2).

## 4. Discussion

Using a large cohort of patients evaluated in the ED for an infectious state, it was found that serum albumin levels used together with the SOFA score improved the prediction of 30-day mortality risk for patients with infections in the ED by 24.3%.

While increasing evidence is available on patients with sepsis and their management, there is far less information available on those with a general infectious state. Wolfertz et al. recently suggested that an infectious state causes more than one-fifth of ED admissions, and that only a small percentage of them (~15%) are already septic on arrival in the ED [1]. Using the SOFA score to identify patients with sepsis is also emphasized in the most recent guidelines [7]. The SOFA score was developed specifically to assess the presence and severity of multi-organ dysfunction in critically ill patients in the ICU, and does not appear to be as accurate for apparently non-critical patients or those in the early stages of infection [12,20,21]. It may not fully reflect the temporal evolution of the disease during the patients’ stay in the ED [22]. It may also be unable to detect at an early stage that a significant proportion of patients with infections who initially appear clinically stable at their initial evaluation in the ED already have microvascular and tissue alterations that can potentially evolve into conditions with organ damage and high mortality [23]. 

As it is widely known, different SOFA scores correspond to different risk of mortality [24]. Only a minority of patients had SOFA scores ≥ 4 (Table 1) and the median SOFA value of deceased patients was 4 (IQR 3–6). Even if the study population was expected to be highly heterogeneous in terms of risk of mortality (consider, for instance, the group of subjects with SOFA scores ≥ 4), the distribution of values, which is markedly different from those of the intensive care unit, indicates that most of our infected patients in the ED setting had relatively low SOFA score values. This partly reflects the inclusion of patients with an infectious state not severe enough to lead to an unstable clinical state.

In this study, the mortality rate was 1.4% for patients with an initial SOFA score of <2, and 3.8% for those with an initial SOFA score of ≤3. Ferreira et al. reported a mortality rate of 0% for patients with low SOFA scores and a non-negligible mortality rate of 7% for individuals with a SOFA score of 3 [24]. Conversely, Monclús Cols et al. reported high mortality rates for patients with low SOFA scores; patients with a SOFA score of 2–3 had mortality rates as high as 43%. These findings emphasize how the SOFA score is inadequate in differentiating the severity of patients with low SOFA scores, and in the early prediction of disease progression in patients admitted to the ED with suspected infections [25,26]. The discriminatory ability of SOFA scores for 30-day mortality in our study (AUROC = 0.802) was almost identical to that reported by Saeed et al. in their large multicenter study of patients with infections admitted to the ED (AUROC = 0.80) [26]. These results confirm that despite good performance, the SOFA score is insufficient for making prognostic assessments crucial to infection management and treatment, and that its safe and effective implementation is needed.

The serum albumin level has recently been identified as a prognostic factor in patients with sepsis in the ICU [7]. Furthermore, albumin has previously been shown to be an effective indicator in identifying patients at risk of a negative outcome, especially those with an infection and an apparently stable clinical state [14]. In addition, the performance of albumin has been investigated in patients with COVID-19 infection or other infections, consistently showing a direct correlation with a more severe infectious state [8].

In a study conducted by Viasus et al. in community-acquired pneumonia, low albumin levels were independently associated with 30-day mortality [27]. A further subsequent study conducted in patients with community-acquired pneumonia showed that the addition of albumin to CURB-65 improved its ability to predict 30-day mortality, with an AUROC of 0.847 (95% CI: 0.755–0.938) [28]. Our choice to evaluate the predictive role of albumin values used together with the SOFA score was hence made, relying on the increasing evidence supporting the role of this marker in predicting negative outcomes in infected patients. Its inclusion in many proposed predictive risk models for these patients reflects its relevance. In their study on post-traumatic patients with sepsis, Lu et al. demonstrated that the predictive ability of a model based on clinical and biochemical markers that incorporated the albumin level (AUROC = 0.799) outperformed the SOFA score (AUROC = 0.698; *p* < 0.001). In another retrospective study on 5240 patients with sepsis, adding other laboratory parameters into the SOFA score improved ability to predict death in 30 days (AUROC = 0.681–0.766) [29,30]. A recent retrospective study on 725 patients with sepsis who were discharged from the hospital found that a serum albumin level of <2.5 mg/dL (odds ratio [OR] = 2.616) and a SOFA score of ≥2 (OR = 2.106) at discharge were independent prognostic factors for one-year mortality, indicating that these two variables may fit well in the prognostic assessment of patients [31].

Serum albumin levels tend to be low on average in studies of patients with sepsis in ICUs, where a longer interval between the start of infection and serum albumin measurement, and especially the current use of large volemic treatments, tend to reduce albumin’s information capacity [32,33]. Conversely, a few studies conducted directly upon patient arrival in the ED found that serum albumin had higher overall averages and showed large differences between patients who died and those who survived at the different time points analyzed, indicating a greater predictive ability when there are more heterogeneous patients [14,34]. These data imply that serum albumin is an earlier marker than the biochemical markers that are already included in the SOFA score.

The predictive improvement resulting from serum albumin values used together with the SOFA score can be explained as follows. The SOFA score incorporates components that are altered in the context of apparent organ damage and suggestive of the onset of organic failure specific to that organ system. This condition appears to occur at a late stage during the pathophysiological process of the infectious state, as organs are able to adapt to damage and utilize their functional reserves. As indicated by the high mortality rates found with organ damage, a medium-to-high SOFA score in the ED suggests an ‘advanced’ condition in which prognostic risk evaluation loses meaning. In patients with a medium–low SOFA score, where organ damage has not yet developed, early microvascular changes may already be present. If left untreated, they evolve into tissue hypoxia and subsequently organic damage typical of sepsis, and may result in high mortality due to sepsis or septic shock [7,8,23,31].

Serum albumin is prematurely lost from the vessels to the interstitium as initial endothelial changes develop due to the cytokine storm in response to infection, resulting in capillary leak syndrome characteristic of the early stages of sepsis [35]. Zdolsek et al. suggested that the decrease in albumin correlates directly with microvascular changes and may indicate a higher risk of tissue hypoxia in these patients [36]. As a result, this possible ability of albumin to be altered in the early vascular stages could overcome the widely emphasized limitation of the SOFA score (that it is not predictive of initial or less severe infectious states) [37]. Including this early vascular marker in an organic dysfunction scoring system could improve the assessment of organ damage.

Our study shows that using a marker of possible microvascular tissue damage together with an organ damage scoring system such as SOFA can improve the complex prognostic assessment of infected patients. Thanks to its role as marker of microvascular tissue damage, serum albumin may predict and correlate with the later development of organ damage [15]. The choice to focus only on albumin was made on the basis of the recent existing literature; however, albumin can be easily obtained and used with the SOFA evaluation at the time of ED admission, proving to be a feasible and effective prognostic biomarker in everyday clinical practice. Further studies evaluating the predictive role of other markers of microvascular injury are needed to confirm and expand our results.

In the present study, we used the NRI to evaluate potential clinical predictive improvement resulting from adding albumin to the SOFA. This technique does not aim to develop a new score by incorporating albumin into the SOFA. This would have required categorizing albumin values according to specific intervals, each with a corresponding score point resulting from a predictive model (which were not used, as the objective of our study was a different one). Instead, we used NRI to evaluate how the presence of albumin alongside SOFA can optimize patient classification, moving patients up or down in risk categories, in both patient outcome and controls. Hence, the provided AUROC does not refer to the accuracy of a new score incorporating albumin into the SOFA, but reflects the overall accuracy of albumin used together with (not within) the SOFA score. For the same reason, we could not identify optimal cut-offs.

This study has a few limitations. It was a single-center study, which could limit the generalizability of the findings. The choice of evaluating albumin used together with SOFA was made a priori, based on previous studies showing the efficacy of this marker in predicting outcomes in patients with infectious states and septic states [8,14,15,20,31,32,33]. It did not account for the therapeutic interventions used after the evaluation in the ED, which could have influenced the primary outcome. Furthermore, the choice of including patients and classifying them as possibly affected by an infection was made by the ED physician. The inability of the patient or next of kin to give consent led to exclusion from the study; this may have led to the exclusion of patients with advanced infectious and septic states with encephalopathy. However, the choice was mandatory based on ethical issues and the Declaration of Helsinki. We did not include COVID-19-positive patients because during the study period, they had different clinical management pathways in our ED [38]. Furthermore, detailed information on the source of infection and administration of antibiotics in the ED was not available, and thus could not be assessed. Finally, it was impossible to adopt a prespecified definition of infection due to the high heterogeneity in these patients.

## 5. Conclusions

Our study, based on a large cohort of patients admitted to the ED for an infection, suggests that using serum albumin values measured at admission together with the SOFA score may improve the prognostic assessment of these patients. Serum albumin, a marker of early microvascular tissue damage, may help to effectively identify infected patients at increased risk of negative outcomes, especially among those who appear stable at ED admission. Further studies are required to confirm our results and to evaluate the possibility of incorporating albumin values into the SOFA score through adequate prognostic modelling. 

## Figures and Tables

**Table 1:** The clinical history and demographic and clinical characteristics of the patients with infectious status enrolled in this study according to their SOFA score category.

Variables	SOFA 0–1	SOFA 2–3	SOFA ≥ 4	*p*-Value
Patients, *n* (%)	493 (51.9)	269 (28.3)	187 (19.7)	
Age, years, median (SD)	59.7 (21.1)	74.5 (17.3)	78.9 (12.1)	<0.001
Sex, *n* (%)				0.083
Female	225 (45.6)	106 (39.4)	70 (37.4)	
Male	268 (54.4)	163 (60.6)	117 (62.6)	
Baseline characteristics, *n* (%)				
Ischemic heart disease	50 (10.2)	53 (19.7)	45 (24.1)	<0.001
Hypertension	203 (41.3)	179 (66.5)	146 (78.1)	<0.001
Atrial fibrillation	46 (9.3)	64 (23.8)	60 (32.1)	<0.001
Diabetes	36 (7.3)	49 (18.2)	37 (19.8)	<0.001
Chronic kidney failure	21 (4.3)	40 (14.9)	51 (27.3)	<0.001
Chronic heart failure	38 (7.8)	51 (19.0)	63 (33.7)	<0.001
Chronic obstructive pulmonary disease	28 (5.8)	39 (14.5)	31 (16.6)	<0.001
Hepatopathy	5 (1.1)	7 (2.6)	14 (7.4)	<0.001
Active tumor	53 (10.8)	29 (10.8)	19 (10.2)	0.979
Stroke or transient ischemic attack	15 (3.0)	25 (9.3)	22 (11.8)	<0.001
Charlson Comorbidity Index, unit, mean (SD)	3.1 (3.1)	4.6 (2.3)	5.7 (2.3)	<0.001
Vital parameters				
Systolic blood pressure (mmHg), mean (SD)	133.8 (22.4)	126.7 (23.6)	112.7 (27.9)	<0.001
Respiratory rate (RR), median (IQR)	18 (16–20)	20 (16–25)	24 (18–30)	<0.001
Peripheral oxygen saturation (%), median (IQR)	97 (95–98)	95 (92–97)	93 (89–96)	<0.001
Heart rate (bpm), median (IQR)	94 (81–107)	97 (82–110)	100 (82–115)	0.100
Temperature (°C), mean (SD)	37.7 (1.1)	37.9 (0.9)	37.7 (1.2)	0.036
Hospitalized, *n* (%)	199 (40.4)	206 (76.6)	169 (90.4)	<0.001
Hospitalized in intensive care unit, *n* (%)	1 (0.2)	10 (3.7)	24 (12.8)	<0.001
NEWS score, point, median (IQR)	2 (1–3)	4 (2–6)	7 (4–10)	<0.001

**Table 2:** The clinical history and demographic and clinical characteristics of the patients with infectious status enrolled in this study, according to the study outcome.

Variables	Alive at 30 Days	Death at 30 Days	*p*-Value
Patients, *n* (%)	865 (91.1)	84 (8.9)	
Age, years, mean (SD)	66.1 (20.4)	84.7 (8.5)	<0.001
Sex, *n* (%)			0.135
Female	359 (41.5)	42 (50)	
Male	506 (58.5)	42 (50)	
Baseline characteristics, *n* (%)			
Ischemic heart disease	125 (14.5)	24 (28.6)	0.002
Hypertension	460 (53.2)	72 (85.7)	<0.001
Atrial fibrillation	142 (16.4)	30 (35.7)	<0.001
Diabetes	103 (11.9)	21 (25.0)	0.002
Chronic heart failure	89 (10.3)	25 (29.8)	<0.001
Chronic kidney failure	132 (15.3)	22 (26.2)	0.019
Chronic obstructive pulmonary disease	89 (10.3)	11 (13.1)	0.454
Hepatopathy	24 (2.8)	3 (3.6)	0.725
Active tumor	87 (10.1)	14 (16.7)	0.092
Stroke or transient ischemic attack	53 (6.1)	10 (11.9)	0.061
Charlson Comorbidity Index, unit, mean (SD)	3.8 (2.9)	6.4 (2.1)	<0.001
Vital parameters			
Systolic blood pressure (mmHg), mean (SD)	129.1 (24.4)	108.4 (26.6)	<0.001
Respiratory rate (RR), median (IQR)	20 (17–26)	26 (18–30)	<0.001
Peripheral oxygen saturation (%), median (IQR)	94 (92–97)	93 (88–96)	<0.001
Heart rate (bpm), median (IQR)	98 (80–110)	106 (89–120)	0.010
Temperature (°C), mean (SD)	37.8 (0.9)	37.4 (1.3)	0.021
Hospitalized, *n* (%)	502 (58.0)	72 (85.7)	<0.001
Hospitalized in intensive care unit, *n* (%)	21 (2.4)	14 (16.7)	<0.001
NEWS score, point, median (IQR)	5 (2–7)	8 (5–11)	<0.001
SOFA score, median (IQR)	1 (0–3)	4 (3–6)	<0.001

**Table 3:** The NRI results obtained by combining serum albumin and the SOFA score. Bold and underlined values indicate patients whose risk prediction improved after including serum albumin to the SOFA score. Bold values correspond to patients whose risk prediction worsened after the inclusion of serum albumin in the SOFA score.

**Patients Dead at 30 Days**					
	**<5%**	**5–15%**	**15–30%**	**>30%**	**Total without Albumin**
**<5%**					7
**5–15%**	3	**4**	**6**	**1**	22
**15–30%**	**3**	12	12	**11**	31
**>30%**		**8**	**1**	23	24
**Total with Albumin**	6	24	19	35	84
**Patients Alive at 30 Days**					
	**<5%**	**5–15%**	**15–30%**	**>30%**	**Total without Albumin**
**<5%**		**23**	**1**		486
**5–15%**	462	109	**18**	**4**	247
**15–30%**	**116**	**32**	46	**15**	103
**>30%**	**10**	**1**	**9**	19	29
**Total with Albumin**	588	165	74	38	865

## Data Availability

Data available on request due to privacy/ethical restrictions.
